# Traits of Exogenous Species and Indigenous Community Contribute to the Species Colonization and Community Succession

**DOI:** 10.3389/fmicb.2018.03087

**Published:** 2018-12-12

**Authors:** Jiemeng Tao, Chong Qin, Xue Feng, Liyuan Ma, Xueduan Liu, Huaqun Yin, Yili Liang, Hongwei Liu, Caoming Huang, Zhigang Zhang, Nengwen Xiao, Delong Meng

**Affiliations:** ^1^School of Minerals Processing and Bioengineering, Central South University, Changsha, China; ^2^Key Laboratory of Biometallurgy, Ministry of Education, Changsha, China; ^3^School of Environmental Studies, China University of Geosciences, Beijing, China; ^4^China Nonferrous Metal Mining (Group) Co., Ltd., Beijing, China; ^5^Hunnan Cotton Science Institute, Changde, China; ^6^State Key Laboratory of Environmental Criteria and Risk Assessment, Chinese Research Academy of Environmental Sciences, Beijing, China

**Keywords:** exogenous species, indigenous community, species colonization, community succession, ecological function

## Abstract

Introducing exogenous species into an environment is an effective method to strengthen ecological functions. The traits of the exogenous species and the indigenous communities, as well as the resistance and subsequent succession of the community to exogenous species, are not well-understood. Here, three different functional consortia were introduced into two extremely acidic systems, leaching heap (LH) and leaching solution (LS), derived from the Zijin copper mine in China. The results showed that the structures of both LS and LH communities were affected by the three consortia, but not all the structural changes were in line with variations of community function. Among the three consortia, only the complementary sulfur oxidizers greatly enhanced copper extraction efficiency of LS (by 50.42%). This demonstrated that functional niche novelty gave exogenous species an advantage to occupy an ecological niche in a complementary manner, thus leading to successful colonization. The resistance to, and subsequent succession by, exogenous organisms varied between the two indigenous communities. More specifically, the LS community with low community diversity and simple composition was susceptible to exogenous species, and the community structural changes of LS were both divergent and irreversible. In comparison, the LH community with greater community diversity and more complex composition was more resistant to exogenous species, with the community structures showing a convergent trend over time despite different species being introduced. Therefore, we propose that diverse communities compete for resources more intensely with exogenous species and resist their introduction, and that communities with complex composition are able to cope with exogenous disturbances.

## Introduction

Introducing exogenous species into an environment is an effective method to strengthen ecological functions. It has been widely employed in soil remediation (Calderon et al., 2017), sewage sludge treatment (Rodriguez-Rodriguez et al., 2014), fermentation process (Haeggman and Salovaara, 2008), and other bio-treatment systems (Wen et al., 2013; Callac et al., 2015). Compared to other systems, in the extremely acidic bioleaching system, it is more common to carry out the introduction or inoculation of exogenous species due to its lower diversity and simpler composition (Panda et al., 2015). Previous studies have demonstrated that introducing exogenous acidophiles, whether a single strain or more complex consortia, into the bioleaching systems improved the bio-oxidization of copper sulfide minerals, removed restricting factors, and changed the original community composition (Liu et al., 2011; Zhang et al., 2015a; Ma et al., 2018). However, not all exogenous species are able to overcome a series of barriers and established in new environments. A systemic understanding of the establishment conditions, community responses to exogenous species and subsequent succession of communities is necessary to reveal and elucidate the effectiveness of exogenous species.

The successful establishment of exogenous species was thought to primarily depend on how they interacted with resources and local indigenous species (Shea and Chesson, 2002). These interactions in turn depended on the traits of both the exogenous species and indigenous community (Low et al., 2015). Functional traits of exogenous species have been examined extensively in an effort to predict their fate (Van Kleunen et al., 2010). It has been reported that high aggressiveness, strong competitiveness, fast growth, and high fecundity were the key behavioral traits to determine the establishment of exogenous species (Nyberg and Wallentinus, 2005; Rehage et al., 2005). Also, many factors of the indigenous community or environment, such as their diversity (van Elsas et al., 2012) and indigenous species traits and interactions (Wei et al., 2015), can affect the inherent susceptibility of the indigenous community and therefore the establishment and survival of exogenous species. The establishment of exogenous species may have consequences not only for the composition of an indigenous community, but also for the functional capacity of the ecosystem as a whole (Amalfitano et al., 2015).

Community succession has always been at the core of community ecology (Prach and Walker, 2011). Observations of microbial community succession after introducing exogenous species are crucial for understanding the susceptibility and stability of ecosystems to external events. The succession of ecological communities can be convergent, divergent, idiosyncratic or other complex forms (Inouye and Tilman, 1995; Li et al., 2016). Previous studies have reported that microbial community successions showed a convergent tendency after different exogenous perturbations (Yannarell et al., 2007; Li and Kajikawa, 2017). However, studies on species introduction showed that when an exogenous strain *At. thiooxidans* A01 or *F. thermophilum* was introduced to an artificial bioleaching consortium, the structure and function of the indigenous consortium showed great divergence from the indigenous communities (Liu et al., 2011; Zhang et al., 2015b). Therefore, the direction of succession that communities may take in response to environmental perturbations, including the introduction of exogenous species, could vary widely.

In this study, we investigate the responses of two *in situ* copper mine communities, leaching heap (LH) and leaching solution (LS) systems in the Zijin Shan copper mine (Fujian province, China), to the inoculation of exogenous species. Instead of artificial systems, measures on *in situ* systems could be more practical and applicable to natural ecosystems. In our previous work, the differences of microbial composition and diversity between these two subsystems have been investigated (Xiao et al., 2016). The ecological functions in bioleaching systems are well-studied and easy to monitor as compared to other natural environments. To explore how exogenous oxidizing microbes affect the community structure and function of LH and LS systems, acidophiles with different oxidative functions (ferrous oxidizers, sulfur oxidizers and ferrous/sulfur oxidizers) were, respectively, introduced into *in situ* microbial communities of LH and LS.

## Materials and Methods

### Minerals and Exogenous Strains

The chalcopyrite ore used in this study was collected from Guangxi, China. After being ground and sieved by a 200-mesh grid, the ore particle diameter was <74 μm. Inductively coupled plasma-atomic emission spectrometry (ICP-AES, PS-6, Baird, USA) analysis revealed that the major elements of the mineral were Fe (28.70%), Cu (33.10%), and S (35.40%). X-ray diffraction (XRD, D/Max 2500, Rigaku, Japan) analysis showed that the mineral sample was mainly composed of chalcopyrite. Then the ore sample was sequentially washed with 2 M HCl, distilled water and pure ethanol. Finally, the mineral sample was dried and reserved in a vacuum desiccator at room temperature until experimental set up.

Six typical acidophilic strains, originally isolated from Dexing copper mine in Jiangxi province, China, were selected to construct three exogenous consortia based on different oxidizing functions. The strains include ferrous-oxidizers (*Leptospirillum ferriphilum* DX2012 and *Ferroplasma acidiphilum* DX2012), sulfur-oxidizers (*Acidithiobacillus caldus* DX2012 and *Acidithiobacillus thiooxidans* DX2012) and ferrous/sulfur-oxidizers (*Acidithiobacillus ferrooxidans* DX2012 and *Sulfobacillus thermosulfidooxidans* DX2012). The detailed culture conditions of each strain are listed in Table S1.

### Preparation of Indigenous Communities

**Figure 1:**
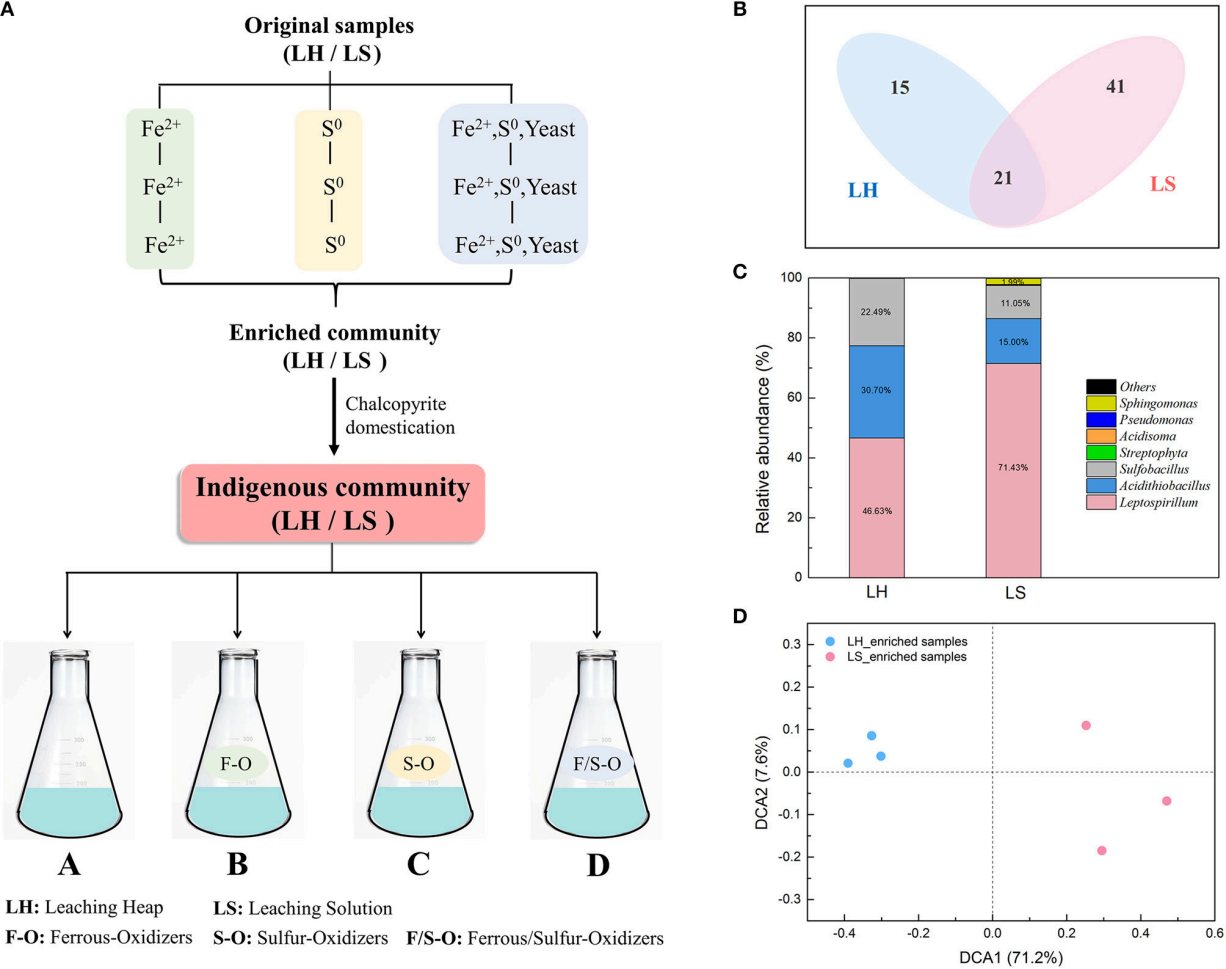
The differences of indigenous microbial communities between LH and LS by chalcopyrite domestication for three generations. **(A)** A rough sketch of the experiment design, **(B)** Distribution of OTUs in LH and LS enrichments, **(C)** Microbial composition at genus level of LH and LS enrichments; **(D)** Detrended correspondence analysis (DCA) of 16S rRNA gene sequencing data between LH and LS enrichments.

Original leaching samples were collected from a leaching heap (LH) and leaching solution (LS) from the Zijin Shan copper mine. The environmental properties of the two leaching systems have been described previously (Xiao et al., 2016). Microorganisms from LH samples were collected by washing the ore surface with sterile dH_2_O (pH 2.0); organisms from LS samples were collected by filtering the solution though a 0.22-μm filter. The collected microorganisms from both samples were, respectively, cultivated in 9K media with three different energy substrates (44.7 g/L FeSO_4_·7H_2_O, 10 g/L S^0^ and 22.4 g/L FeSO_4_·7H_2_O + 5 g/L S^0^ + Yeast extract (0.2 g/L)) at pH 2.0 and 30°C for 3–5 days with three replicates each time (Figure 1A). The initial inoculum concentrations of LH and LS were 10% wt/vol and 20% vol/vol, respectively. Inoculum concentrations were then adjusted to 20% vol/vol for the second and third enrichment by transferring the last cultures. After three successive generations, microbial cultures of the three different media were mixed in a flask for LH and LS, respectively. The mixed cultures were domesticated in 500-mL shake flasks with 100 mL 9K medium and 2% (w/v) chalcopyrite (as mentioned above) for 30 days for three successive transfers. Then, indigenous leaching microbial communities were obtained by filtering, centrifuging, and washing the mineral residues of the third enriched culture of leachates. Sample solutions from each culture were kept stationary for 30 min and the supernatant was filtered through a 0.22-μm filter. The settled sediments in the flask were re-suspended and shaken on a vortex generator by adding 20 mL of sterile basal media for several times until no cells could be detected by microscopy. Finally, cells were collected by centrifuging the mixed supernatant at 12,000 × g for 10 min. Subsequently, total DNA from each sample was extracted and sequenced by 16S rDNA to analyze the microbial community.

### Experiments Setup

The experiments were conducted in 500-mL shake flasks, each containing 250 mL 9K medium and 2% pulp density (w/v) of chalcopyrite at pH 2.0 (Figure 1A). Before inoculation, acid pre-leaching, with 18 M H_2_SO_4_, was performed every 12 h for 72 h, to control pH at 2.0. After 72 h, microbial enrichments of indigenous leaching communities for both LH and LS were, respectively, inoculated with an initial cell density of 1.0 × 10^7^ cells/mL. After an adaptive phase, different exogenous consortia with a cell density of 1.0 × 10^8^ cells/mL were introduced to the leaching systems prior to the log phase of LH (3rd day) and LS (6th day). Based on different energy utilization strategies of the exogenous strains, bioleaching experiments were classified into four systems: Indigenous community (System A), Indigenous community + ferrous-oxidizers of *L. ferriphilum* DX2012 and *F. acidiphilum* DX2012 (System B), Indigenous community + sulfur-oxidizers of *A. caldus* DX2012 and *A. thiooxidans* DX2012 (System C) and Indigenous community + ferrous/sulfur-oxidizers of *A. ferrooxidans* DX2012 and *S. thermosulfidooxidans* DX2012 (System D). Meanwhile, the abiotic control was set up for both LH and LS. The experiments were carried out in triplicate at 30°C and 175 rpm for 33 (LH) and 36 (LS) days. During the experiment, loss by evaporation and sampling was supplemented by adding, respectively, distilled water and 9K medium periodically. Here, we focused on copper extraction as a model ecosystem function due to the leaching substrate of chalcopyrite. The copper concentration was assayed based on the color reaction with bis (cyclohexanone) oxalyldihydrazone (Chimpalee et al., 1995).

### Analysis of Community Structure by 16S rDNA Sequencing and RT-qPCR

Five milliliter of bioleaching solution was collected from each flask on days 0, 3, 9, 15, 21, 27, and 33 for LH systems and on days 0, 6, 12, 18, 24, 30, and 36 for LS systems. Samples were centrifuged at 2,000 × g for 2 min to separate the supernatant from the bottom sediment and transferred the supernatant into a new centrifuge tube. Then the bottom ore sample was re-suspended and shaken on a vortex generator by adding 5.0 mL of sterile basal media several times until no cells could be detected by microscopy. All the re-suspended solution was transferred into the above centrifuge tube. Finally, cells were collected by centrifuging the mixed supernatants at 12,000 × g for 10 min. Subsequently, total DNA from each sample was extracted with the TIANamp® Bacteria DNA kit (Tiangen Biotech, Co. Ltd., Beijing, China) following the manufactures' manual and was checked by 1% (w/v) agarose gel electrophoresis.

16S rDNA sequencing was performed as previously described (Tao et al., 2016). PCR reactions were conducted to amplify the V4 hyper variable region of the 16S rDNA (~250 nucleotides) with the primer set 515F (5'-GTGCCAGCMGCCGCGGTAA-3') and 806R (5'-GGACTACHVGGGTWTCTAAT-3') combined with Illumina adapter sequences and barcodes (Caporaso et al., 2012). Sequencing libraries were constructed using 200 ng of each purified PCR product and sequencing was performed on an Illumina MiSeq machine (Illumina, San Diego, CA, USA) with a 500-Cycles kit (2 × 250 bp paired-ends) at the Key Laboratory of Biometallurgy of the Ministry of Education in Central South University. Sequencing data was processed on an in-house galaxy pipeline developed in the lab of Dr. Jizhong Zhou (http://zhoulab5.rccc.ou.edu/), as previously described (Li et al., 2017). To summarize, raw sequences with perfect matches to barcodes were split into sample libraries and trimmed using Btrim (Kong, 2011) with the threshold of QC higher than 20 over a five bp window size and the minimum length of 100 bp. Subsequently, forward and reverse reads were assembled to complete sequences by Flash (Magoc and Salzberg, 2011). Finally, chimeric sequences were removed, sequences were clustered into Operational Taxonomic Units (OTUs) with 97% sequence similarity, and an OTU table without singletons was generated using UPARSE (Edgar, 2013). Taxonomic assignment was carried out through RDP classifier (Wang et al., 2007) with a minimal 50% confidence estimate. To eliminate the bias caused by different sequencing depths, samples were rarefied at 10,000 sequences per sample. All sequences were classified into 289 OTUs, 138 OTUs for LH and 233 OTUs for LS. The raw data from the high-throughput sequencing has been submitted to the Sequence Read Archive (SRA) of NCBI, and the project ID is SRP 141342.

Real-time quantification polymerase chain reaction (RT-qPCR) was performed to detect the different strains of *Acidithiobacillus* spp. and further to reveal community succession. RT-qPCR primers for the three *Acidithiobacillus* strains used in this study (Table S2) were designed by Primer Premier 5.0 and synthesized by BioSune (BioSune Biological Engineering Technology & Services Co. Ltd., Shanghai, China). Before RT-qPCR, conventional PCR was performed and the products were diluted serially from 10^4^ to 10^10^ copies/mL and amplified by RT-qPCR to construct standard curves. The detailed procedure for RT-qPCR was performed with the iCycler iQ Real-time PCR detection system (Bio-Rad Laboratories Inc., Hercules, USA) as previously described (Ma et al., 2017a).

### Colonization Assay of the Exogenous Species

The whole course of bioleaching could be divided into three consecutive phases, the strain adaptive-growing phase, the rapidly increasing phase and the stationary phase (Ma et al., 2017a). Introduction was regarded as “successful” if the exogenous species were able to persist in the leaching systems up to the stationary phase. The rapidly increasing phase was the most important period during which the acidophilic bacteria grew fast and made full use of oxidative function to dissolve minerals, thus only species that were competitive enough were able to colonize and survive in this phase. Exogenous consortia were introduced to the native leaching systems prior to the start of the log phase of LH and LS, with approximately 12 days left before transiting to the stationary phase. Twelve days were enough for communities to achieve a stable state of abundance after introduction, and therefore a long enough time period to gauge whether the exogenous species had become integrated into the resident community (Rivett et al., 2018). Before the experiments were initiated, the 16S rRNA gene of the six exogenous strains were amplified using the primer set 27F (5'-AGAGTTTGATCCTGGCTCAG-3') and 1492R (5'-AGAGTTTGATCCTGGCTCAG-3') and were then sequenced by BioSune (BioSune Biological Engineering Technology & Services Co. Ltd., Shanghai, China). The colonized (exogenous) species were determined by assessing which sequences were not present in the original “indigenous” consortia and comparing them to the known exogenous sequences. First, new species were identified by comparing OTU tables and selecting the new OTUs after introduction. New OTUs must (i) not be detected in all systems before exogenous species introduction; (ii) appear as new species after introducing exogenous species, and (iii) be detected in the corresponding experimental groups during the whole bioleaching process. OTUs whose total number was < 10 or only appeared once in the three replicates were removed. After selecting new OTUs, their sequences were extracted and aligned with the sequences of the six exogenous strains to confirm their taxa. The relative abundance of the new species was used to determine their colonization and survival.

### Data Analysis

All calculations and statistical analyses for the microbial community were carried out in R v. 3.4.0 (R Foundation for Statistical Computing, Vienna, Austria). Community analyses were performed based on a relative abundance of OTUs in each sample. Alpha diversity was evaluated by Shannon-Weiner's index (*H*), the Simpson index (*D*), Pielou evenness (*J*), and Simpson evenness (*Si*) to describe the microbial community's diversity. Principle component analysis (PCA) was carried out with the vegan package (Dixon, 2003) using weighted Unique Fraction of branches shared (UniFrac) distances (Lozupone et al., 2011). Non-parametric tests including analysis of similarity (ANOSIM), permutational multivariate analysis of variance (PERMANOVA) and unweighted paired-group method with arithmetic means (UPGMA) were performed based on a Bray-Curtis distance matrix to test the differences in microbial communities among different treatments over time. The graphs and charts were generated by Origin 9.0 or R v. 3.4.0. One-way analysis of variance (ANOVA) followed by Tukey's test was used to determine the differences among the different treatments at the same time in SPSS 22.0 (SPSS Inc., Chicago, USA). A repeat-measures two-way ANOVA was carried out with time, treatment and their interactions as explaining variables to access the effect of the main factors on the copper concentration and alpha diversity. A *p*-value of < 0.05 was considered as significant. All experiments were performed at least three times.

## Results

### The Differences of Indigenous Microbial Community Between LH and LS by Chalcopyrite Domestication

After chalcopyrite domestication for three generations (Figure 1A), microbial systems in LH and LS were maintained in a steady state condition. The Venn diagram (Figure 1B) shows that 21 OTUs were shared by both LH and LS samples, and the unique OTUs in LH and LS were 15 and 41, respectively. The taxonomic composition of the microbial community (Figure 1C) showed that the native samples of LH and LS were dominated by genera of *Leptospirillum, Acidithiobacillus* and *Sulfobacillus*. The proportion of *Leptospirillum* in LS (71.43%) was much higher than that in LH (46.63%), while *Acidithiobacillus* and *Sulfobacillus* were significantly more abundant in LH (30.70% and 22.49%, respectively) than in LS (15.00% and 11.05%, respectively). Correspondingly, the structures of LH and LS were also different. The DCA plot (Figure 1D) showed that the samples in LH were separated from those in LS. The analysis of similarity (ANOSIM) indicated that the distance between LH and LS was significantly (global *R* = 0.845, *p* = 0.001) higher than that between replicates within the same system. Four diversity indexes including the Shannon index (*H*), the Simpson index (*D*), Pielou evenness (*J*) and Simpson evenness (*Si*) (Table 1) in LH were significantly (*p* < 0.05) higher than that in LS.

**Table 1:** Differences between the alpha diversity indexes of indigenous communities based on 16S rDNA sequencing data.

**Native communities**	**Alpha diversity indexes**			
	**Shannon index (*H*)**	**Simpson index (*D*)**	**Pielou evenness (*J*)**	**Simpson evenness (*Si*)**
LH	1.504 ± 0.069*a*	0.707 ± 0.018*a*	0.474 ± 0.012*a*	0.143 ± 0.006*a*
LS	0.964 ± 0.074*b*	0.461 ± 0.024*b*	0.293 ± 0.065*b*	0.071 ± 0.034*b*

*LH, microbial community of leaching heap; LS, microbial community of leaching solution. Different letters following the results indicate the difference is statistically significant (p < 0.05)*.

### Ecological Functioning Performance in LH and LS Systems by Introducing Different Microbial Consortia

**Figure 2:**
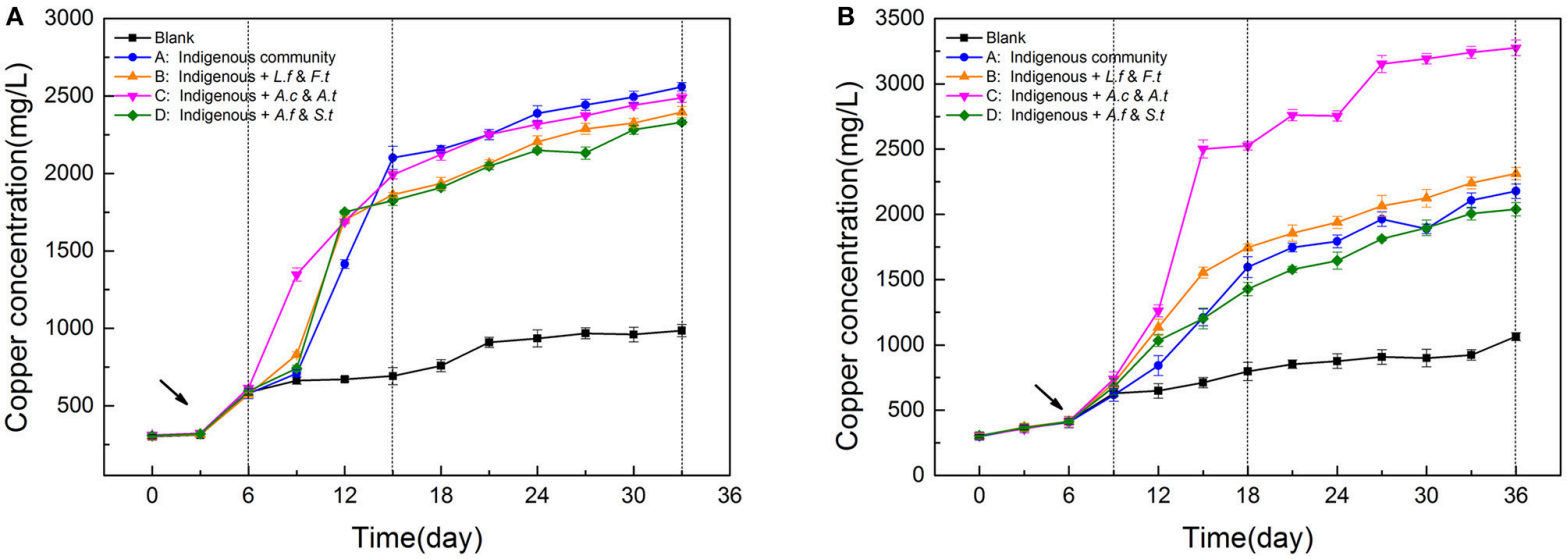
Variation of Copper concentration during leaching process of LH **(A)** and LS **(B)** following introduction of different exogenous consortia. LH, microbial community of leaching heap; LS, microbial community of leaching solution.

The dissolved copper concentration during the bioleaching process of LH and LS are shown in Figure 2. The process could be divided into three phases: the strain adaptive-growing phase (SAG, 0–6th d for LH and 0–9th d for LS), the rapidly increasing phase (RIC, 6–15th d for LH and 9–18th d for LS) and the stationary phase (STA, 15–33th d for LH and 18–36th d for LS) (Ma et al., 2017a). Strains with different types of energy utilization were introduced prior to the RIC phase.

The variation of copper concentration showed that exogenous consortia had a stronger influence on LS (low diversity) than on LH (high diversity), and that different microbial functional traits could affect the leaching process in various ways. The copper concentration variation of the four treatments in LH (Figure 2A) showed a similar pattern during the bioleaching process, with the exogenous species having only a slight effect on copper extraction efficiency by the end of the experiment. Compared with LS, LH presented a shorter SAG phase. Consistent with this, the dissolution rate of chalcopyrite in LH was faster in the beginning 15 days and the end copper efficiency in LH (38.7%) was significantly higher (*p* < 0.05, ANOVA following Tukey test) than that in LS (32.9%) when there were no exogenous species, indicating that the diverse community was more efficient in resource utilization. In contrast, LS was more susceptible to exogenous species (Figure 2B), in which system C showed the greatest capacity in dissolving copper from minerals. The copper concentration of the four systems achieved maximum differences on Day 15 with values of 1211 ± 64, 1554 ± 69, 2501 ± 79, and 1203 ± 41 mg/L, respectively. As the STA phase began, the dissolution rate of copper ion increased slowly owing to a higher jarosite yield. Then, the copper concentration of system C was the first to achieve a steady state (3153 ± 65 mg/L), on day 27, and reached the highest copper concentration (3276 ± 59 mg/L) in the end. Compared to the indigenous system, exogenous sulfur oxidizers in LS increased the copper extraction rate to 49.48%, which indicated that exogenous sulfur oxidizers could establish themselves in the new environment and possessed a stronger leaching ability for chalcopyrite than the other exogenous species. Repeat-measures two-way ANOVA (Table S3) showed leaching time had significant effects on the copper concentration in both LH and LS systems, but that the different exogenous strain treatments only had obvious effects on the LS systems.

### Different Community Structures Between LH and LS Systems by Introducing Different Microbial Consortia

As an important member of the bioleaching system, the dynamics of acidophilic microorganisms in a community were closely related to the dissolution process of minerals. Therefore, microbial community succession was investigated by 16S rDNA sequencing and RT- qPCR. A total of 10,000 rarified 16S rRNA gene sequences were obtained for each sample and clustered into 289 OTUs at a 97% similarity threshold.

**Figure 3:**
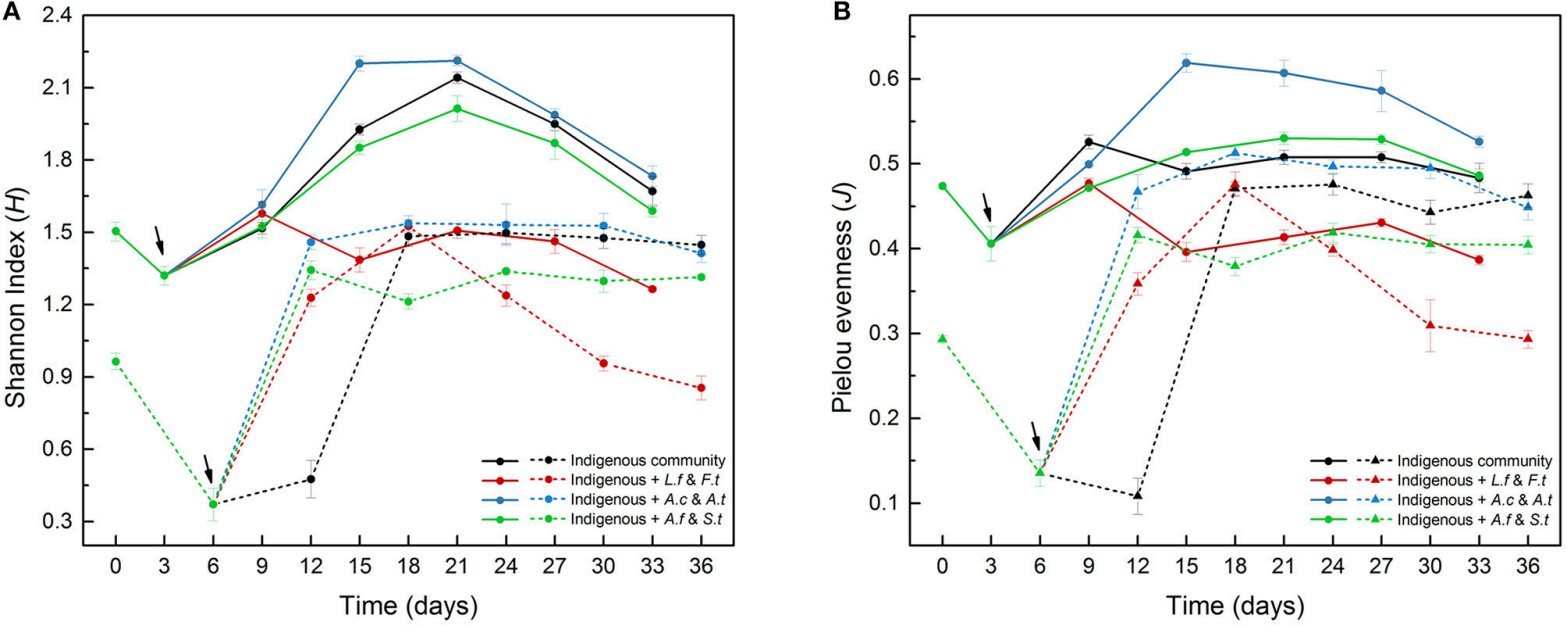
The α-diversity indexes, including Shannon diversity index **(A)** and Pielou evenness **(B)**, of four different treatments in LH (solid lines) and LS (dashed lines) during the leaching process. LH, microbial community of leaching heap; LS, microbial community of leaching solution.

**Figure 4:**
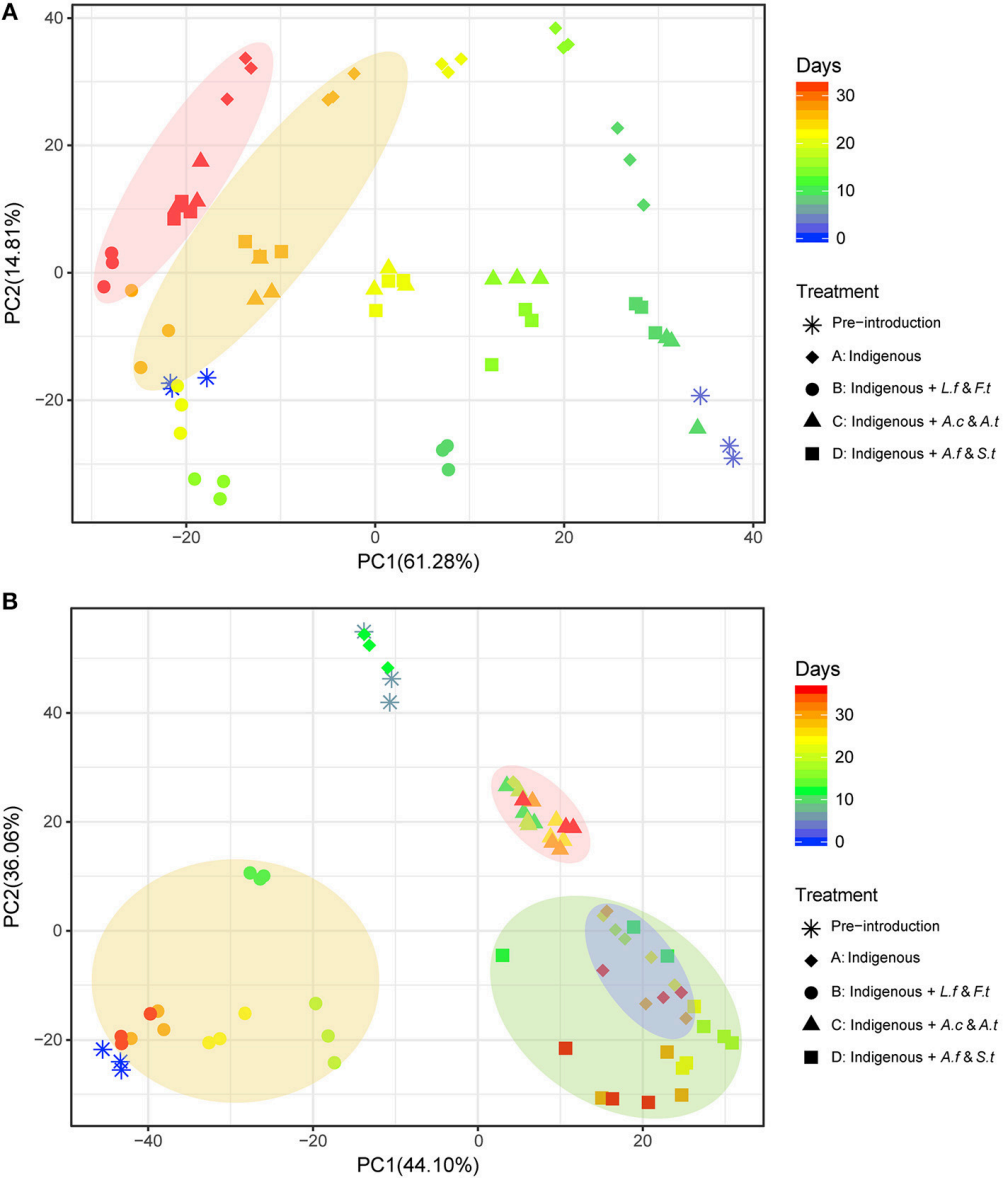
Principal component analysis (PCA) of microbial community succession in LH **(A)** and LS **(B)** by different exogenous consortia, different shapes indicate different treatments and colors of shapes indicate the variation of leaching time. The shaded regions represent leaching time in LH **(A)** and different systems in LS **(B)**. LH, microbial community of leaching heap; LS, microbial community of leaching solution.

The variation of Shannon diversity and Pielou evenness in LH (Table S4) and LS (Table S5) showed similar tendencies with time (Figure 3). Due to the intensified competition among acidophilic cells for insufficient energy at the start of the experiment, these two indexes decreased rapidly from Day 0 to Day 3 (LH) or Day 6 (LS), which was consistent with the distribution variation in the PCA plot (Figure 4). Compared to LH, the introduction of exogenous species had more obvious effects on LS with lower community diversity. For example, the diversity indexes in LS treatments (B, C and D) were significantly (*p* < 0.05) higher than in system A from Day 6 to Day 18, but in LH systems, only system C showed a significant (*p* < 0.05) variation. Similar trends were also observed for Simpson index and Simpson evenness in LH (Table S4) and LS (Table S5). At the same time, Repeat-measures two-way ANOVA (Table S3) showed the significant effects of leaching time and treatments on the four diversity indexes.

As shown in Figure 4, exogenous consortia caused different effects on the two indigenous systems. Before the exogenous strains were added, samples on Day 3 for LH and Day 6 for LS were both distant from the samples on Day 0. For LH systems (Figure 4A), after introducing exogenous strains, samples from the same system were scattered across time points. Generally, all samples in LH systems showed clear convergence over time and were closely clustered at day 33. For LS treatments (Figure 4B), after introducing exogenous species, samples from the same treatment appeared to group very well with each other regardless of time variation, and sample distribution differed significantly across different treatments. PERMANOVAS results for LH (Table S6) and LS (Table S7) showed that leaching time had more significant effects on the LH system and treatment had a more significant effect on the LS system. Moreover, the unweighted paired-group method with arithmetic means (UPGMA) cluster tree also clustered the samples in LH (Figure S1A) and LS (Figure S1B) according to leaching time and leaching treatments, respectively. The results also indicated a similar distribution and that samples from LH tended to converge with time but samples in LS clustered well depending on treatments.

### Microbial Community Succession in LH and LS Systems

**Figure 5:**
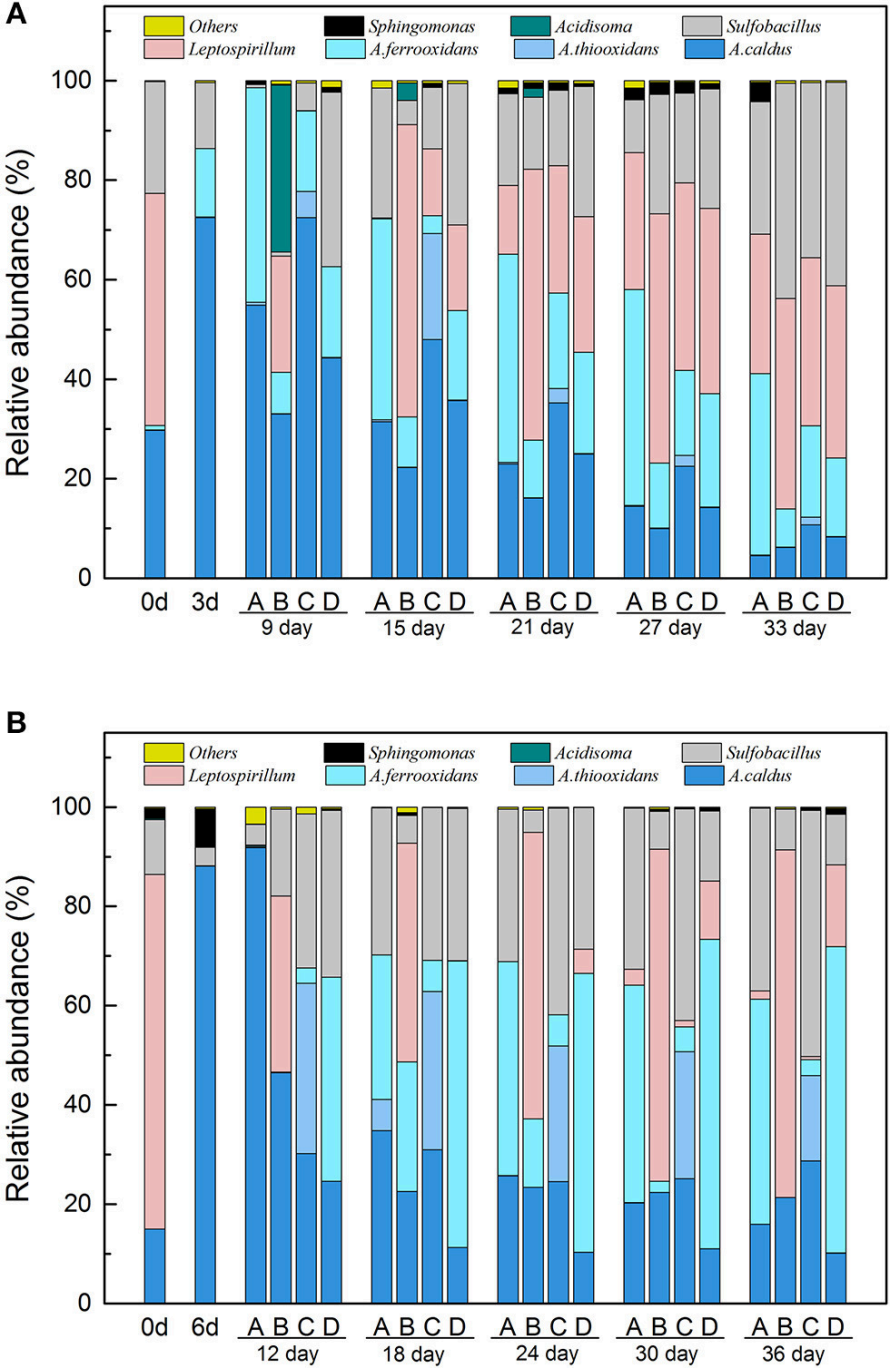
The microbial community succession of acidophilc cells in the four systems of LH **(A)** and LS **(B)**. LH, microbial community of leaching heap; LS, microbial community of leaching solution. A, Indigenous community; B, Indigenous + *L. ferriphilum* & *F. acidiphilum*; C, Indigenous + *A. caldus* & *A. thiooxidans*; D, Indigenous + *A. ferrooxidans* & *S. thermosulfidooxidans*.

Given the results of both 16S rDNA sequencing and RT-qPCR, the differences of key bacteria succession in LH and LS are shown in Figure 5 and Table S8. The taxonomic composition of the microbial community showed different succession patterns as induced by different types of exogenous species. In LH systems (Figure 5A), *A. caldus* increased dramatically and predominated by Day 3. After exogenous species introduction, the microbial composition in B and C showed differences from community A. In system B, *Leptospirillum* predominated the system throughout, and there were more *A. thiooxidans* in system C. Sulfur oxidizers accounted for a large proportion of organisms in the RIC phase with *Leptospirillum* and *Sulfobacillus* becoming important genera in the latter stage. In LS systems (Figure 5B), the community's initial composition was primarily of *Leptospirillum* and *A. caldus*, with proportions of 72.63 and 17.77%, respectively, *A. caldus* increased dramatically to 85.33% by Day 6. After introduction of exogenous species, the taxonomic composition showed obvious alterations. For example, *Leptospirillum* became the predominant species of system B by Day 18, *At. thiooxidans* accounted for a much higher proportion in system C and *At. ferrooxidans* was more abundant in system D, indicating that exogenous species might perform a successful colonization of the indigenous community. In the RIC phase, *Acidithiobacillus* spp. showed a gradual tendency to increase in the four treatments as the concentration of copper ions in the leachates increased rapidly. While transitioning into the STA phase, the proportion of *Acidithiobacillus* spp. remained at a relatively stable level, but *Leptospirillum* and *Sulfobacillus* continued to increase until the end of the experiment.

### Successful Colonization and Survival of Exogenous Species in LH and LS

**Figure 6:**
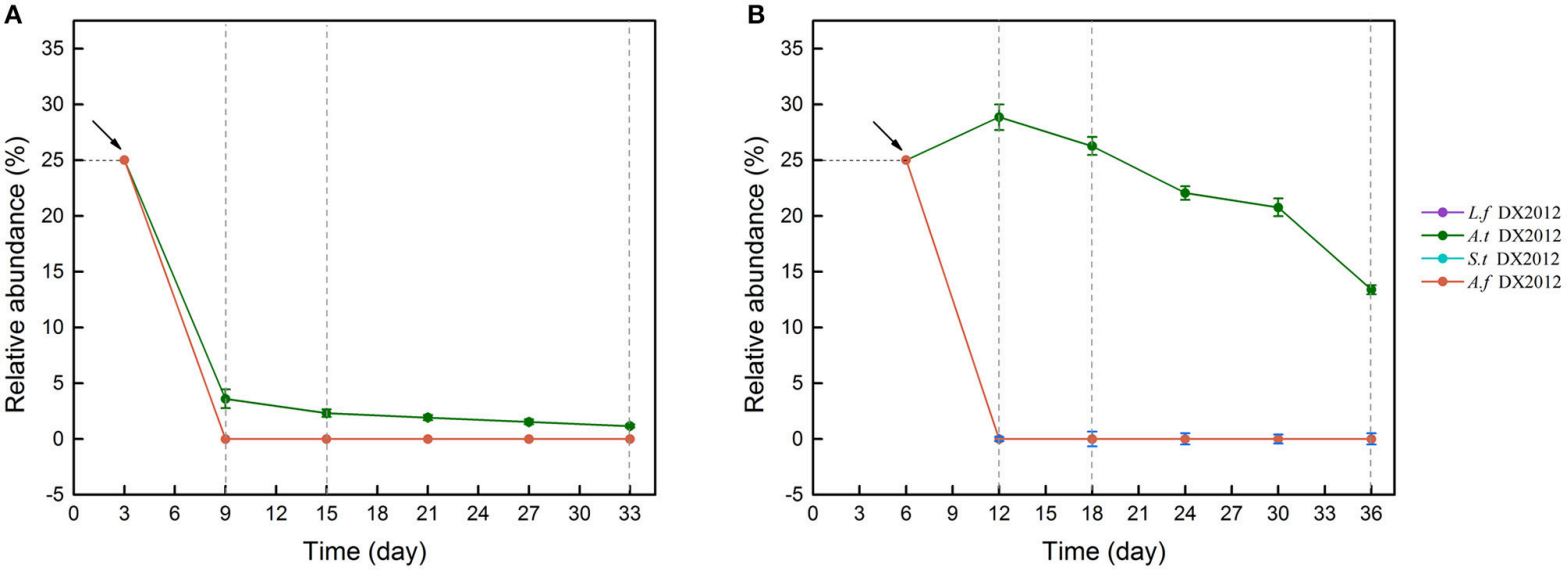
Exogenous species survival in LH **(A)** and LS **(B)** by introducing different oxidizers over time. The detailed information for the survivors is shown in Table S9. LH, microbial community of leaching heap; LS, microbial community of leaching solution.

Sequence alignment (Figure S2) indicated OTU_177, OTU_212, OTU_102 and OTU_121 were identified as *Leptospirillum ferriphilum* DX2012, *Acidithiobacillus thiooxidans* DX2012, *Acidithiobacillus ferrooxidans* DX2012 and *Sulfobacillus thermosulfidooxidans* DX2012, respectively, but the OTUs representing *Acidithiobacillus caldus* DX2012 and *Ferroplasma acidiphilum* DX2012 were not detected in this experiment. The 16S rRNA sequences of the strains and the representative sequences of the four OTUs are shown in Table S9. The colonization and survival of exogenous species were calculated and analyzed (Figure 6). The amount of each exogenous species accounted for 25% of the corresponding system. In LH (Figure 6A), a progressive decline was observed for the four exogenous bacteria from Day 3 onward. After Day 9, no exogenous species could be detected except for a small number of *At. thiooxidans* DX2012. In LS (Figure 6B), a rapid decline was also observed for *L. ferriphilum* DX2012, *S. thermosulfidooxidans* DX2012 and *A. ferrooxidans* DX2012. In contrast, the relative abundance of *At. thiooxidans* DX2012 increased during the first 6 days of introduction and maintained a high proportion (>25%) during the RIC phase. Afterwards, the proportion of *At. thiooxidans* DX2012 decreased slightly but maintained a detectable and high abundance (>15%) until the end of the experiment.

All the results indicated that LS was more susceptible to exogenous species than LH and the successful colonization of exogenous species could make an influence on the structure and function of indigenous communities.

## Discussion

As an open ecosystem, the microbial and geochemical simplicity of the bioleaching system makes it an ideal model for species introduction tests. However, due to the low pH and high concentration of toxic elements in this system, exogenous microbes are strictly filtered and excluded. First, given the highly specialized environment, we chose microorganisms from a similar acidic environment, the Dexing copper mine in China. Second, in line with recent research showing that a close exotic-native phylogenetic relatedness could increase the chance of successful colonization due to niche pre-adaptation (Li et al., 2015), exogenous species were restricted to common genera, *Leptospirillum, Ferroplasma, Acidithiobacillus*, and *Sulfobacillus* spp. (Méndezgarcía et al., 2014). In addition, a recent study showed that introducing several species simultaneously could elevate overall colonization success (Rivett et al., 2018). Therefore, based on functional categories, two ferrous oxidizers (*L. ferriphilum* DX2012 and *F. acidiphilum* DX2012), two sulfur oxidizers (*A. caldus* DX2012 and *A. thiooxidans* DX2012), and two ferrous/sulfur oxidizers (*A. ferrooxidans* DX2012 and *S. thermosulfidooxidans* DX2012) were chosen as exogenous consortia, respectively. Here, two leaching subsystems, LH and LS, were used as indigenous systems in which to carry out the experiments. Considering the activity and adaption of original samples, chalcopyrite domestication of LH and LS was performed for three generations. As expected, the domestication process generated greater diversity, evenness, composition, and structural differences between the two communities (Figure 1). Under different indigenous communities, introducing exogenous species could potentially result in different outcomes.

The probability of successful colonization by exogenous species was partly related to the susceptibility of the indigenous community (Amalfitano et al., 2015). Similar to microbial invasion, exogenous species colonization is also a process with the four consecutive stages of introduction, establishment, spread, and impact (Mallon et al., 2015). During each phase, there may exist large (unsurmountable) or small (surmountable) barriers deriving from the indigenous community. Many factors could potentially influence the susceptibility of a community to exogenous species, including habitat suitability and disturbance, community structure, and resource supply (Jiang and Morin, 2010). In our study, consortia with the same oxidative function showed significantly different colonization abilities in LH and LS systems, especially for sulfur oxidizers, which established successfully in the LS system but could not been detected in the LH system. Except for possessing the same traits as the exogenous species, the underlying reasons might be attributed to the different characteristics of the indigenous community, such as diversity, composition and interaction networks. The diversity level (indexes of *H, D, J*, and *Si*) of the LH community was significantly higher than that in LS (Table 1). Under conditions of limited resources, diverse communities compete for resources more intensely than simple ones (Mallon et al., 2015, 2016), which could prevent species establishment and subsequent growth (Jousset et al., 2011; van Elsas et al., 2012). Therefore, the LH community might pose a stronger resistance to exogenous microbes. Another dimension that also needs to be considered is microbial community composition. Recent studies have reported that the phylogenetic composition of indigenous communities acts as a biological barrier against exogenous *L. monocytogenes* (Vivant et al., 2013). Similarly, the distribution of OTUs at genus level (Figure 1) showed great differences between LH and LS systems, suggesting that the taxonomic composition of the indigenous community may also contribute to the barrier effects. The community stability and function could be enhanced by complementary dominant species to protect against exogenous species (Allan et al., 2011). The abundance and composition of sulfur oxidation was so high in LH (Figure 1C) that there was little room left and few resources for exogenous oxidizers. Besides, in the natural environment, leaching heap microorganisms have formed a very cohesive ecological network over long periods, but microbial interactions in leaching solution are constantly changing because of its fluid nature (Xiao et al., 2016). Therefore, a highly diverse and connected community of LH might be more resistant to exogenous species.

The effects of exogenous species were also specific to the type of bacteria (Harkins et al., 2013) and how “invasive” they are. In our study, three types of exogenous consortia showed significantly different effects on the LS community. The sulfur consortium was found to be the most successful at colonizing, while the ferrous and ferrous/sulfur consortia could not establish themselves in the LS community (Figure 6). The differences might be caused by the intrinsic traits of the exogenous consortia and the interactions between the consortia and native microbes. The fate of an introduced species partly depends on how novel its functional niche is in the indigenous community (Thuiller et al., 2010; Castro-Diez et al., 2014). The proportion of ferrous oxidizers in LS (>71.43%) was high (Figure 1), which resulted in a higher niche overlap between the resident bacteria and exogenous species and increased the probability of competitive exclusion of the exogenous species (Wei et al., 2015). In contrast, more niches were left for sulfur oxidization by the low abundance of *Acidithiobacillus*, so exogenous sulfur oxidizers could break the biotic resistance and occupy suitable niches, potentially leading to large impacts on the function of the indigenous community. Inoculation of exogenous species has been applied to many bioleaching systems to enhance bio-oxidization functions (Zhang et al., 2015a; Ma et al., 2018). Similarly, our study showed that exogenous sulfur oxidizers greatly enhance the chalcopyrite extraction rate of the LS system by modifying the community consumption rates of available resources. Interestingly, *Acidithiobacillus thiooxidans* seemed to possess a stronger ability to establish themselves than *Acidithiobacillus caldus* (Figures 5, 6) although they hold similar ecological functions. This was also in accordance with previous statements that communities with more sulfur oxidizers were more efficient (Zeng et al., 2016; Ma et al., 2017a,b).

Even if microbial communities are sensitive to disturbances, such as biological inoculation and physical stimuli, some of them may still be resilient and return to their pre-disturbance composition after a short time (Allison and Martiny, 2008). Community succession can be an important reflection on the ability of various systems to address exogenous perturbations. Accordingly, an interesting phenomenon between the variation of LH and LS structures occurred. The structures of treated communities in LS were subjected to a more serious fluctuation than LH after introducing exogenous species, while the community structures in LH systems showed a tendency to converge over time, but the impacts of newly introduced species on LS systems were divergent (Figure 4), suggesting the LH system was able to mitigate the effects of exogenous species. It has been reported that the impacts of biological inoculation could be mitigated through eradicating the exogenous species or through the development of new control mechanisms by the native species (Li and Kajikawa, 2017). The community succession direction might be related to community traits and individual characteristics of community members (Zhou et al., 2014). Here, the higher biodiversity and network complexity of LH made it more resistant to exogenous species. Moreover, microorganisms in LH had faster growth rates (Xiao et al., 2016); thus, if their abundance was suppressed by a disturbance, they had the potential to recover more quickly (Allison and Martiny, 2008). Additionally, more genetically diverse communities are more likely to contain taxa with complementary response traits (Elmqvist et al., 2003; Tilman et al., 2006) and possess the ability for rapid compensatory growth after a disturbance (Flöder et al., 2010), thus enhancing the resilience of LH systems.

The ecological impacts of exogenous species are usually assessed by comparing species fitness, community structure and finally the ecological function (Castro-Diez et al., 2014; Kumschick et al., 2015). Using community function as an indicator of exogenous impacts may help to bridge the gap between impacts on community structure and impacts on ecosystem processes (Castro-Diez et al., 2016). As a disturbance, introducing exogenous species caused changes to community structure, such as microbial composition (Figure 5), diversity (Figure 3), and community distribution (Figure 4). However, a more basic question is whether the variation of community structure always results in the changes of community function or whether microbial composition shifts matter to ecosystem processes. A previous study focusing on a soil microbial community showed that structural changes did not influence the ecological function in the microcosms (Wertz et al., 2010). Similarly, only system C in LS showed significant functional differences in the bioleaching process (Figure 2) in spite of the structural changes in all treated communities (Figure 3), indicating that not all the structural changes were in line with the variation of community function. There are three reasons to address this question. First, the exogenous species only caused a fluctuation, but did not spread or become established, in the indigenous community. The fluctuation was able to affect the community structure to a degree but could not further alter the community function. Second, the exogenous species might be functionally redundant with the taxa already present in the indigenous community. For instance, *Leptospirllum* spp. was already so abundant in LS that the addition of exogenous ferrous oxidizers could not enhance this function further. Additionally, taxa within the communities may function differently but resulted in the same process rate when combined at the community level. In our study, we focused on the copper extraction rate as a model ecosystem function. In fact, the ecological functions in the bioleaching systems are diverse and complex. Combined metagenomic and transcriptomic analyses can provide great assistance to reveal the potential ecological functions and adaptive mechanisms of acidophilic microbes (Chen et al., 2015). Therefore, further studies will focus on the functional partitioning of microbes by the technologies of metagenome and transcriptome, which we hope will supplement and verify the present conclusions.

In conclusion, our experiment showed that traits of both exogenous species and the indigenous community contributed to the colonization or rejection of exogenous species and the community succession. The simple community might be susceptible to microbial inoculation and species with functional niche novelty could occupy a complementary ecological niche and increase functional abilities. The colonized exogenous species might enhance the ecological function of the indigenous community. The indigenous community might show a convergent trend if the effects by the exogenous species were not strong enough.

## Author Contributions

DM, XL, and HY helped design this study and contributed material essential for the study. CQ and XF designed the sampling strategy, collected the original samples from the copper mine, and helped to finish the experiment. CH and ZZ provided great assistance at the sampling site. LM, YL, HL, and NX were all involved in finishing the experiment. JT conducted the data analysis and wrote the first draft of the manuscript and all authors made substantial contributions to the revisions of the manuscript.

### Conflict of Interest Statement

The authors declare that the research was conducted in the absence of any commercial or financial relationships that could be construed as a potential conflict of interest.
