# Supplementary material for: Traits of Exogenous Species and Indigenous Community Contribute to the Species Colonization and Community Succession
Source: Front Microbiol. 2018 Dec 12;9:3087. doi: 10.3389/fmicb.2018.03087 (PMC6299020; doi:10.3389/fmicb.2018.03087)
Supplement: Supplementary file 1 [file Data_Sheet_1.pdf]

Supplemental Information for:

Traits of exogenous species and indigenous community contribute to the species colonization and community succession

Jiemeng Tao[1,2], Chong Qin[1,2], Xue Feng[1,2], Liyuan Ma[3], Xueduan Liu[1,2], Huaqun Yin[1,2], Yili Liang[1,2], Hongwei Liu[1,2], Zhigang Zhang[4], Caoming Huang[5], Nengwen Xiao[6] and Delong Meng[1,2*]

*Correspondence:

Delong Meng: Delong.meng@gmail.com;

Tel: +86(731)88830546

Fax: +86(731)88830546

SI: Figures

(a)

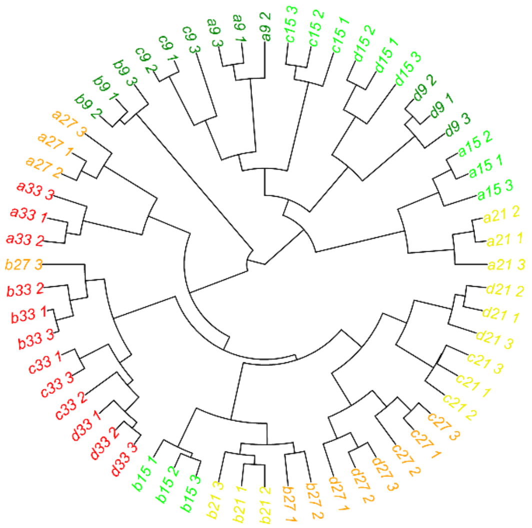

(b)

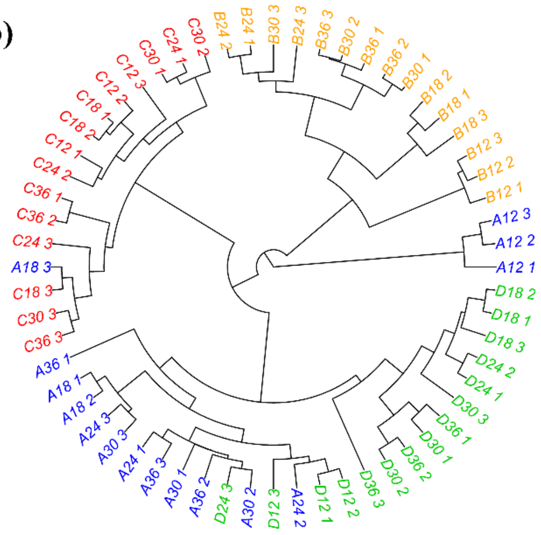

Figure S1 Unweighted paired group method with arithmetic mean (UPGMA) tree by Bray-Curtis distances between microbial community in LH system (a) and LS system (b) by introducing different exogenous consortia, colors indicate different leaching time in LH and indicate different treatments in LS. Different letters represent different treatments and the number following the letter was the time during leaching process. **a/A**: indigenous community in LS/LH; **b/B**: indigenous community + *L. ferriphilum* DX2012& *F. acidiphilum* DX2012 in LH/LS; **c/C**: indigenous community + *A.caldus* DX2012 & *A.thiooxidans* DX2012 in LH/LS; **d/D**: indigenous community + *A.ferrooxidans* DX2012 & *S.thermosulfidooxidans* DX2012 in LH/LS.

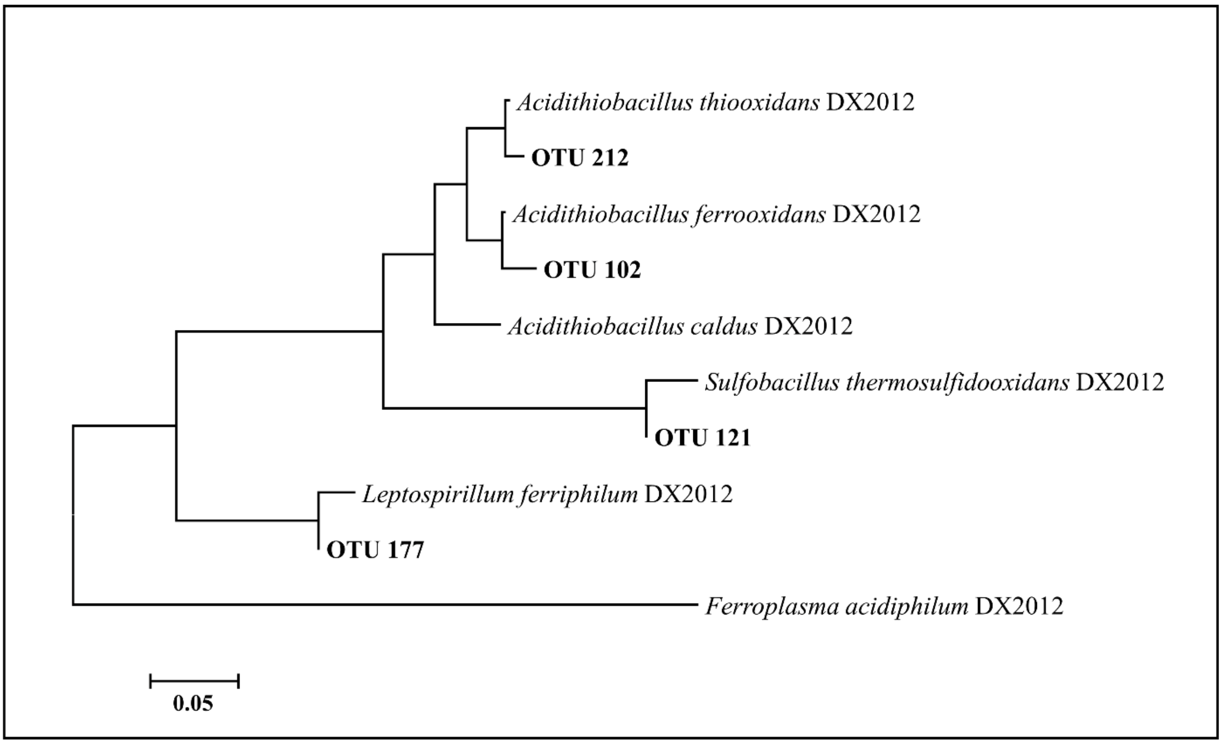

Figure S2 Phylogenetic tree of the selected OTUs and exogenous species based on 16S rDNA sequences

SI: Tables

Table S1 Characteristic features and physiological properties of the six exogenous strains used in this study

**Species (strain)**	**Energy type**	**Optimal growth conditions**		
		Media (g/L)	pH	Temp (℃)
Acidithiobacillus caldus (DX2012)	Sulfur oxidizer	9K + S (10)	2.0	45
Acidithiobacillus thiooxidans (DX2012)	Sulfur oxidizer	9K + S (10)	2.0	30
Acidithiobacillus ferrooxidans (DX2012)	Ferrous/sulfur oxidizer	9K + $FeSO_4$·7H2O (22.4) + S (5)	2.0	30
Sulfobacillus thermosulfidooxidnas (DX2012)	Ferrous/sulfur oxidizer	9K + $FeSO_4·7H_2O$ (22.4) + S (5) + yeast extract (0.2)	1.6	45
Leptospirillum ferriphilum (DX2012)	Ferrous oxidizer	9K + $FeSO_4·7H_2O$ (44.7)	1.6	40
Ferroplasma thermophilum (DX2012)	Ferrous oxidizer	9K +$FeSO_4·7H_2O$ (44.7) + yeast extract (0.1)	1.0	45

The 9K basal medium consists of the following basal salts (g/L): $(NH_4)_2SO_4$ (3.0), K_2HPO_4 (0.5), KCl (0.1), $Ca(NO_3)_2$ (0.01), $MgSO_4·7H_2O$ (0.5).

Table S2 The designed primers of the three *Acidithiobacillus* strains for RT-qPCR

Target species	Primer names	Primer sequences (5'-3')	Amplicon length (bp)
At. caldus	soxX1-S	CAGTATTCCACCCATCAACG	114
	soxX1-A	ACTCCACCTGGCAAGACAT	
At. thiooxidans	Sqr-S	GCTCGGCAGCCTCAATAC	136
	Sqr-A	GGTCGGACGGTGGTTACTG	
At. ferrooxidans	rus-S	ACAAGGGATTCGGTCATAGTTT	153
	rus-A	CCGTCGGATGCCAGGTAAA	

Table S3 Two-way ANOVA analysis of copper concentration and diversity indexes in different treatments during the leaching process. The effects of Time, Treatment and Time × Treatment are indicated by *p* values; *p*-values highlighted in gray show significance ($p < 0.05$).

		Two-way ANOVA		
		Time	Treatment	Time & Treatment
LH	Copper concentration	<0.001	0.287	0.076
	Shannon Index (*H*）	0.017	0.009	0.008
	Simpson Index (*D*)	0.015	0.022	0.020
	Pielou evenness (*J*)	0.022	0.002	0.009
	Simpson evenness (*Si*)	0.010	0.005	0.005
LS	Copper concentration	<0.001	0.034	0.003
	Shannon Index (*H*)	0.007	0.008	0.002
	Simpson Index (*D*)	0.018	0.010	0.006
	Pielou evenness (*J*)	0.005	0.016	0.011
	Simpson evenness (*Si*)	0.002	0.011	0.009

Table S4 The variation of alpha diversity indexes in the four LH systems over time. Statistical significant difference of one way ANOVA analysis is indicated by different letters ($p < 0.05$).

Time (days)	Microbial communities	Shannon Index (*H*)	Simpson Index (*D*)	Pielou evenness (*J*)	Simpson evenness (*Si*)
0		1.504±0.069	0.707±0.018	0.474±0.012	0.143±0.007
3		1.321±0.139	0.569±0.056	0.406±0.030	0.092±0.009
9	A	1.517±0.025a	0.724±0.010a	0.525±0.013a	0.202±0.015a
	B	1.578±0.050a	0.740±0.011a	0.476±0.021a	0.142±0.023b
	C	1.613±0.163a	0.662±0.047b	0.499±0.027a	0.118±0.002b
	D	1.524±0.047a	0.712±0.009a	0.471±0.011a	0.137±0.001b
15	A	1.927±0.022b	0.808±0.003a	0.491±0.009b	0.103±0.010c
	B	1.385±0.050c	0.608±0.016b	0.396±0.010c	0.077±0.001c
	C	2.199±0.030a	0.835±0.010a	0.618±0.010a	0.174±0.011a
	D	1.850±0.027b	0.793±0.003a	0.513±0.001b	0.131±0.003b
21	A	2.141±0.023a	0.834±0.001a	0.507±0.016b	0.089±0.008c
	B	1.506±0.050b	0.657±0.023b	0.413±0.016c	0.076±0.003c
	C	2.212±0.022a	0.848±0.001a	0.606±0.005a	0.172±0.004a
	D	2.013±0.053a	0.829±0.010a	0.530±0.020b	0.132±0.014b
27	A	1.949±0.027a	0.797±0.008a	0.507±0.032b	0.107±0.024b
	B	1.462±0.099b	0.671±0.028b	0.430±0.037c	0.102±0.012b
	C	1.986±0.026a	0.799±0.006a	0.586±0.011a	0.168±0.011a
	D	1.869±0.065a	0.790±0.012a	0.528±0.037b	0.139±0.025a
33	A	1.670±0.061a	0.773±0.015a	0.483±0.017a	0.139±0.009a
	B	1.264±0.005b	0.633±0.001b	0.386±0.005c	0.103±0.005b
	C	1.733±0.043a	0.751±0.012a	0.526±0.006a	0.149±0.009a
	D	1.589±0.025a	0.725±0.006a	0.486±0.008a	0.138±0.010a

A: indigenous community; **B**: indigenous community + *L. ferriphilum* DX2012& *F. acidiphilum* DX2012; **C**: indigenous community + *A.caldus* DX2012 & *A.thiooxidans* DX2012; **D**: indigenous community + *A.ferrooxidans* DX2012 & *S.thermosulfidooxidans* DX2012. Different small letters after the numbers in the same column under the different densities indicated significant differences at the level of 0.05.

Table S5 The variation of alpha diversity indexes in the four LS systems over time. Statistical significant difference of one way ANOVA analysis is indicated by different letters ($p < 0.05$).

Time (days)	Microbial communities	Shannon Index (*H*)	Simpson Index (*D*)	Pielou evenness (*J*)	Simpson evenness (*Si*)
0		0.964±0.074	0.461±0.024	0.293±0.065	0.071±0.034
6		0.370±0.248	0.194±0.172	0.135±0.116	0.076±0.062
12	A	0.476±0.277b	0.154±0.099b	0.108±0.051c	0.020±0.014c
	B	1.228±0.036a	0.641±0.015a	0.359±0.023b	0.091±0.014b
	C	1.459±0.101a	0.724±0.021a	0.467±0.050a	0.163±0.058a
	D	1.343±0.059a	0.688±0.016a	0.416±0.059a	0.130±0.051a
18	A	1.482±0.071a	0.710±0.022a	0.471±0.018a	0.148±0.008b
	B	1.525±0.047a	0.704±0.014a	0.476±0.035a	0.139±0.035b
	C	1.537±0.073a	0.734±0.028a	0.513±0.040a	0.189±0.040a
	D	1.213±0.072b	0.602±0.020b	0.379±0.021b	0.103±0.013c
24	A	1.497±0.044a	0.713±0.020a	0.475±0.036a	0.151±0.034a
	B	1.237±0.045b	0.600±0.028b	0.398±0.011b	0.112±0.005b
	C	1.531±0.086a	0.721±0.027a	0.497±0.068a	0.168±0.055a
	D	1.339±0.003b	0.629±0.030b	0.419±0.025b	0.110±0.014b
30	A	1.475±0.042a	0.700±0.033a	0.443±0.028ab	0.121±0.029ab
	B	0.956±0.131c	0.498±0.039b	0.309±0.036c	0.091±0.002b
	C	1.527±0.052a	0.711±0.014a	0.495±0.026a	0.158±0.016a
	D	1.298±0.135b	0.589±0.054b	0.405±0.040b	0.100±0.009b
36	A	1.447±0.040a	0.694±0.010a	0.463±0.028a	0.144±0.029a
	B	0.854±0.050b	0.461±0.035c	0.293±0.023b	0.101±0.018b
	C	1.413±0.114a	0.661±0.044a	0.449±0.030a	0.128±0.011a
	D	1.314±0.099a	0.597±0.042b	0.404±0.021a	0.097±0.016b

A: indigenous community; **B**: indigenous community + *L. ferriphilum* DX2012& *F. acidiphilum* DX2012; **C**: indigenous community + *A.caldus* DX2012 & *A.thiooxidans* DX2012; **D**: indigenous community + *A.ferrooxidans* DX2012 & *S.thermosulfidooxidans* DX2012. Different small letters after the numbers in the same column under the different densities indicated significant differences at the level of 0.05

Table S6 Significance tests on the effects of time and treatments on the microbial community structures in LH systems by using permutational multivariate analysis of variance (PERMANOVAS), calculated with Bray distance. The results are indicated by *p* values; *p*-values highlighted in gray show significance ($p < 0.05$).

		3 day	9 day	15 day	21 day	27 day	33 day
Time	0 day	0.001	0.003	0.002	0.008	0.003	0.001
	3 day		0.096	0.003	0.006	0.001	0.001
	9 day			0.017	0.001	0.001	0.001
	15 day				0.091	0.001	0.001
	21 day					0.029	0.001
	27 day						0.001
		System B	System C	System D			
Treatment	System A	0.001	0.012	0.006			
	System B		0.005	0.004			
	System C			0.183			

Table S7 Significance tests on the effects of time and treatments on the microbial community structures in LS systems by using permutational multivariate analysis of variance (PERMANOVAS), calculated with Bray distance. The results are indicated by *p* values; *p*-values highlighted in gray show significance ($p < 0.05$).

		6 day	12 day	18 day	24 day	30 day	36 day
Time	0 day	0.001	0.003	0.003	0.005	0.003	0.004
	6 day		0.077	0.001	0.002	0.005	0.002
	12 day			0.062	0.018	0.025	0.023
	18 day				0.927	0.531	0.444
	24 day					0.834	0.800
	30 day						0.967
		System B	System C	System D			
Treatment	System A	0.001	0.001	0.001			
	System B		0.001	0.001			
	System C			0.001			

Table S8 The differences of bacteria abundance in different systems over time. Statistical significant difference of one way ANOVA analysis is indicated by different letters ($p < 0.05$).

	Time	Treatment	One-way ANOVA						
			A.caldus	*A.thiooxidans*	*A.ferrooxidans*	*Leptospirillum*	*Sulfobacillus*	*Acidisoma*	*Sphingomonas*
LH	0 day		-	-	-	-	-	-	-
	3 day		-	-	-	-	-	-	-
	9 day	System A	b	b	a	b	c	b	a
		System B	c	-	c	a	c	a	b
		System C	a	a	b	b	b	b	b
		System D	c	-	b	b	a	b	a
	15 day	System A	b	-	a	c	a	b	b
		System B	c	-	c	a	c	a	b
		System C	a	a	d	b	b	b	a
		System D	b	-	b	b	a	b	b
	21 day	System A	b	-	a	c	b	b	ab
		System B	c	-	c	a	b	a	ab
		System C	a	a	b	b	b	b	a
		System D	b	-	b	b	a	b	b
	27 day	System A	b	-	a	b	c	-	a
		System B	b	-	b	a	a	-	a
		System C	a	a	b	b	b	-	a
		System D	b	-	b	b	a	-	b
	33 day	System A	b	-	a	b	b	-	a
		System B	b	-	c	a	a	-	b
		System C	a	a	b	b	b	-	b
		System D	ab	-	b	b	a	-	b
LS	0 day		-	-	-	-	-	-	-
	6 day		-	-	-	-	-	-	-
	12 day	System A	a	b	c	b	c	-	a
		System B	b	-	c	a	b	-	b
		System C	c	a	b	b	a	-	b
		System D	c	-	a	b	a	-	a
	18 day	System A	a	b	b	b	a	-	b
		System B	b	-	b	a	b	-	a
		System C	a	a	c	b	a	-	c
		System D	c	-	a	b	a	-	b
	24 day	System A	a	-	b	c	b	-	b
		System B	a	-	c	a	c	-	b

		System C	a	a	d	c	a	-	a
		System D	b	-	a	b	b	-	b
	30 day	System A	a	-	b	c	b	-	b
		System B	a	-	d	a	d	-	b
		System C	a	a	c	d	a	-	b
		System D	b	-	a	b	c	-	a
	36 day	System A	bc	-	b	c	b	-	c
		System B	b	-	d	a	c	-	b
		System C	a	a	c	c	a	-	a
		System D	c	-	a	b	c	-	a

Table S9 16S rDNA sequences of the six exogenous species and the V4 hyper variable region sequences of the selected OTUs corresponding to each species

Invader species	16S ribosomal RNA gene of the invaders	V4 hyper variable region sequences of the selected OTUs corresponding to each invader
L. ferriphilum DX2012	TGCAGTCCGACGTGAAAGGGGAGCAATCCCCCGGTAGGGTGGCAAACGGGTGAGTAAGACATGGGTGATCTGCCCTG<br>GAGATGGGGATATCCCTCCGAAAGGGGGGCAATACCGAATAGTATCCGGTTCCGTGAAGGGGGCCGGGGAAAGGG<br>AGGCCTCTGGTACAAGCTTCCGCTCCTGGATGAGCCCATGGCCCATCAGCTAGTTGGTAGGGTAAAGGCCTACCAAGG<br>CGACGACGGGTAGCTGGTCTGAGAGGACAACCAGCCACACTGGCACTGAGACACGGGCCAGACTCCTACGGGAGGCA<br>GCAGTGAGGAATATTGCGCAATGGGGGCAACCCTGACGCAGCAACGCCGCGTGTGGGAAGAAGGCTTTCGGGTTGTA<br>AACCACTTTTGCCCGGGACGAAAGGGGGGGCCTGAATAAGGTCACCCGATGACGGTACCGGGAGAATAAGCCACGGC<br>TAACTCTGTGCCAGCAGCCGCGGTAAGACAGAGGTGGCAAGTGTTGTTCGGAGTTACTGGGCGTAAAGAGTCTGTAGG<br>TGGTCTGTCAAGTCTTTGGTGAAAGGCCGTGGCTTAACCATGGGAATGCCAAAGAGACTGGCAGACTGGAGGCTGGG<br>AGAGGGTAATGGAATTTCTGGTGTAGCGGTGAAATGCGTAGATATCAGAAGGAAGGCCGGTGGCGAAGGCGGCTTCC<br>TGGAACAGACCTGACACTGAGAGACGAAAGCGTGGGGAGCAAACAGGATTAGATACCCTGGTAGTCCACGCCCTAAA<br>CGATGGGTACTAAGTGTGGGAGGGTTAAACCTCCCGTGCCGCAGCCAACGCAGTAAGTACCCCGCCTGGGGAGTACG<br>GCCGCAAGGTTGAAACTCAAAGGAATTGACGGGGGCCCGCACAAGCGGTGGTGCATGTGGTTTAATTCGACGCAACG<br>CGAAGAACCTTACCTGGGCTTGACATGCCGCGAGTAGGGAACCGAAAGGGGACCGACCGGTTCAATCCGGAAGCGGA<br>ACAGGTGCTGCATGGCTGTCGTCAGCTCGTGCCGTGAGGTGTTGGGTTCAGTCCCGCAACGAGCGCAACCCTCGCCCT<br>CTGTTGCCACCGGGTCATGCCGGGCACTCTGAGGGGACTGCCAGCGACAAGTTGGAGGAAGGAGAGGATGACGTCAA<br>GTCATCATGGCCCTTATGCCCAGGGCCACACACGTGCAACAATGGCCGGTACAGACGGAAGCAAGACCGAGAGGTGG<br>AGCAAATCCGAGAAAGCCGGTCCCAGTTCGGATTGAGGTCTGCAACTCGACCTCATGAAGTCGGAATCGCTAGTAATC<br>GCGTATCAGCACGACGCGGTGAATACGTTCCCGGGCCTTGTACACACCGCCCGTCACACCACGAAAGTCTGTTGTACC<br>TGAAGTCGGTGCCCCAACCGGAAACGGAGGAGCC	**>OTU_177**<br>GACAGAGGTGGCAAGTGTTGTTCGGAGTTACTGG<br>GCGTAAAGAGTCTGTAGGTGGTCTGTCAAGTCTTT<br>GGTGAAAGGCCGTGGCTTAACCATGGGAATGCCA<br>AAGAGACTGGCAGACTGGAGTATGGGAGAGGGTG<br>ATGGAATTCCAGGTGTAGCGGTGAAATGCGTAGA<br>TATCAGAAGGAAGGCCGGTGGCGAAGGCGGCTTC<br>CTGGAACAGACCTGACACTGAgagaCGAAAGCGTG<br>GGGAGCAAACAGG

F.acidiphilum DX2012	CCTGCGAGTCAGGTATCGTAAGATGCCGGCAAACTGCTCAGTAACACGTGGATAATCTAACCTTGAGTAAGGATAAC<br>TTCGGGAAACTGAAGGTAATACCTTATAATTGCTTAAAACTGGAATGTTTTTGCAATAAAAGTTACGACGCTCAAGGA<br>TGAGTCTGCGACCTATCAGGTAGTAGGTGGTGTAATGGACCACCTAGCCTCAGACGGGTACGGGCCCTGGGAGGGGT<br>AGCCCGGAGATGGACTCTGAGACATAAGTCCAGGCCCTACGGGGCGCAGCAGGCGCGAACACTGTGCAATGCGCGAA<br>AGCGCGACACGGGGAGCTTGAGTGTCTTGGCATAGCCAAGACTTTTCTCATTCCTAAAAAGCATGAGGAATAAGTGCT<br>GGGTAAGACGGGTGCCAGCCGCCGCGGTAACACCCGCAGCACGAGTAGTGGTCACTTTTATTGAGCCTAAAGCGTTCG<br>TAGCCGGTTTTGTAAATCTTCAGATAAAGCCTGAAGCTTAACTCCAGAAAGTCTGAAGAGACTGCAAGACTTGAGATC<br>GGGTGAGGTTAAACGTACTTTCAGGGTAGGGGTAAAATCCTGTAATCCCGGAAGGACGACCAGTGGCGAAAGCGTTT<br>AACTAGAACGAATCTGACGGTAAGGAACGAAGGCTAGGGTAGCAAACCGGATTAGATACCCGGGTAGTCCTAGCTGT<br>AAACATTGCCCATTTGATGTTGCTTTTCCGTTGAGGGAAGGCAGTGTCGGAGCGAAGGTGTTAAATGGGCCGCTTGGG<br>AAGTATGGTCGCAAGACTGAAACTTAAAGGAATTTGGCGGGGGAGCACCGCAACGGGAGGAATGTGCGGTTTAATTG<br>GATTCAACGCCGGAAAACTCACCGGGAACGACCTGTGCATGAGAGTCAACCTGACGAGCTTACTCGATAGCAGGAGA<br>GGTGGTGCATGGCCGTCGTCAGCTCGTACCGTAGGGCGTTCACTTAAGTGTGATAACGAGCGAGACCCACATCTTTAA<br>TTGCAAATGTATATGAGAATATGCATGCACTTTAGAGAAACCGCCAGCGCTAAGCTGGAGGAAGGAGTGGTCGACGG<br>CAGGTCAGTACGCCCCGAATTTCCCGGGCTACACGCGCATTACAAAGAACGGGACAATACGTTGCAACCTCGAAAGA<br>GGAAGCTAATCGCGAAACCCGTCCATAGTTAGGATTGAGGGCTGTAACTCGCCCTCATGAATCTGGATTCCGTAGTAA<br>TCGCGTC	None

A.caldus DX2012	GCAGTCGGACGGCAGCAGGTCCTTCGGGATGCTGGCGAGTGGCGGACGGGTGAGTAATGCGTAGGAACCTATCCTTTT<br>GTGGGGGACAACCCAGGGAAACTTGGGCTAATACCGCATAAGCCCTGAGGGGGAAAGCGGGGGATCTTCGGACCTCG<br>TGCTGAAGGAGGGGCCTACGTCCGATTAGCTAGTTGGTGGGGTAAAGGCCTACCAAGGCGACGATCGGTAGCTGGTCT<br>GAGAGGACGACCAGCCACACTGGGACTGAGACACGGCCCAGACTCCTACGGGAGGCAGCAGTGGGGAATTTTTCGCA<br>ATGGGGGCAACCCTGACGAAGCAATGCCGCGTGGATGAAGAAGGCCTTCGGGTTGTAAAGTCCTTTCGTGGGGGACG<br>AAAAGGCGGATCCGAATACGGTCTGCTATTGACGTGAACCCAAGAAGAAGCACCGGCTAACTCCGTGCCAGCAGCCG<br>CGGTAATACGGAGGGTGCGAGCGTTAATCGGAATTACTGGGCGTAAAGGGCGCGTAGGCGGTGGGTTACGTCTGCCG<br>TGAAATCCCCGGGCTCAACCTGGGAATGGCGGTGGAAACGGGCTGACTGGAGTATGGGAGAGGGTGATGGAATTCCA<br>GGTGTAGCGGTGAAATGCGTAGAGATCTGGAGGAACACCAGTGGCGAAGGCGGTCACCTGGCCCAATACTGACGCTG<br>AGGCGCGAAAGCGTGGGGAGCAAACAGGATTAGATACCCTGGTAGTCCACGCCCTAAACGATGGATACTGGATGTTT<br>GGCGCCTTAGGTGCTGAGTGTCGTAGCTAACGCGATAAGTATCCCGCCTGGGAAGTACGGCCGCAAGGTTAAAACTCA<br>AAGGAATTGACGGGGGCCCGCACAAGCGGTGGAGCATGTGGTTTAATTCGATGCAACGCGAAGAACCTTACCTGGGC<br>TTGACATGTCCGGAACCCTGCAGAGATGTGGGGGTGCCCTTCGGGGAATCGGAACACAGGTGCTGCATGGCTGTCGTC<br>AGCTCGTGTCGTGAGATGTTGGGTTAAGTCCCGCAACGAGCGCAACCCTTGTTCCTAGTTGCCAGCGGTTCGGCCGGG<br>CACTCTAGGGAGACTGCCGGTGACAAACCGGAGGAAGGTGGGGATGACGTCAAGTCCTCATGGCCTTTATGTCCAGG<br>GCTACACACGTGCTACAATGGTGCGTACAGAGGGAAGCCAAGCCGCGAGGTGGAGCAGACCCCAGAAAGCGCGTCGT<br>AGTTCGGATTGCAGTCTGCAACTCGACTGCATGAAGTCGGAATCGCTAGTAATCGCGGATCAGCATGCCGCGGTGAAT<br>ACGTTCCCGGGCCTTGTACACACCGCCCGTCACACCATGGGAGTGGATGGTACCAGAAGCCGTTAGCCTAACCTTCGG<br>GGGGGCGACG	None

A.thiooxidans DX2012	TGCAGTCGAACGGTAACAGGTCTTCGGATGCTGACGAGTGGCGGACGGGTGAGTAATGCGTAGGAATCTGTCTTTGAG<br>TGGGGGACAACCCAGGGAAACTTGGGCTAATACCGCATAAGCCCTGAGGGGGAAAGCGGGGGATCTTCGGACCTCGC<br>GCTGGAAGAGGAGCCTACGTCTGATTAGCTAGTTGGTAGGGTAAAGGCCTACCAAGGCGACGATCGGTAGCTGGTCT<br>GAGAGGACGACCAGCCACACTGGGACTGAGACACGGCCCAGACTCCTACGGGAGGCAGCAGTGGGGAATTTTTCGCA<br>ATGGGGGCAACCCTGACGAAGCAATGCCGCGTGAATGAAGAAGGCCTTCGGGTTGTAAAGTTCTTTCGTGGAGGACG<br>AAAAGGTGGGTGCTAATATCGCCTGCTGTTGACGTGAATCCAAGAAGAAGCACCGGCTAACTCCGTGCCAGCAGCCG<br>CGGTAAGACGGGGGGGGCAAGCGTTAATCGGAATCACTGGGCGTAAAGGGTGCGTAGGCGGTGCATTAGGTCTGTCG<br>TGAAATCCCCGGGCTCAACCTGGGAATGGCGGTGGAAACCGGTGTACTAGAGTATGGGAGAGGGGTGGTGGGAATTC<br>CAGGGTGTAGCGGTGAAATGCGTAGAGATCTGGAGGAACATCAGTGGCGAAGGCGGCCACCTGGCCCAATACTGACG<br>CTGAGGCACGAAAGCGTGGGGAGCAAACAGGATTAGATACCCTGGTAGTCCACGCCCTAAACGATGAATACTAGATG<br>TTTGGTGCCAAGCGTACTGAGTGTCGTAGCTAACGCGATAAGTATTCCGCCTGGGAAGTACGGCCGCAAGGTTAAAAC<br>TCAAAGGAATTGACGGGGGCCCGCACAAGCGGTGGAGCATGTGGTTTAATTCGATGCAACGCGAAGAACCTTACCTG<br>GGCTTGACATGTCTGGAATCCTGCAGAGATGCGGGAGTGCCCTTCGGGGAATCAGAACACAGGTGCTGCATGGCTGTC<br>GTCAGCTCGTGTCGTGAGATGTTGGGTTAAGTCCCGCAACGAGCGCAACCCTTGTCCTTAGTTGCCAGCGGTTCGGCC<br>GGGCACTCTAGGGAGACTGCCGGTGACAAACCGGAGGAAGGTGGGGATGACGTCAAGTCCTCATGGCCTTTATGTCC<br>AGGGCTACACACGTGCTACAATGGCGCGTACAGAGGGAAGCCAAGCCGCGAGGTGGAGCAGACCCCAGAAAGCGCG<br>TCGTAGTTCGGATTGCAGTCTGCAACTCGACTGCATGAAGTCGGAATCGCTAGTAATCGCGGATCAGCATGCCGCGGT<br>GAATACGTTCCCGGGCCTTGTACACACCGCCCGTCACACCATGGGAGTGGATTGTACCAGAAGCCGTTAGCCTAACCT<br>TCGGGAGGGCGA	>**OTU_212**<br>GACGTAGGGGGCAAGCGTTAATCGGAATCACTGG<br>GCGTAAAGGGTGCGTAGGCGGTGCATTAGGTCTGT<br>CGTGAAATCCCCGGGCTCAACCTGGGAATGGCGG<br>TGGAAGCCGGTGTACTAGAGTATGGGAGAGGGTG<br>GTGGAATTCCAGGTGTAGCGGTGAAATGCGTAGA<br>GATCTGGAGGAACATCAGTGGCGAAGGCGGCCAC<br>CTGGCCCAATACTGACGCTGAGGCACGAAAGCGT<br>GGGGAGCAAACAGG

A.ferrooxidans DX2012	TGCAGTCGAACGGTAACAGGTCTTCGGATGCTGACGAGTGGCGGACGGGTGAGTAATGCGTAGGAATCTGTCTTTTAG<br>TGGGGGACAACCCAGGGAAACTTGGGCTAATACCGCATGAGCCCTGAGGGGGAAAGCGGGGGATCTTCGGACCTCGC<br>GCTAAGGGAAGAGCCTACGTCTGATTAGCTAGTTGGTAGGGTAAAGGCCTACCAAGGCGACGATCAGTAGCTGGTCT<br>GAGAGGACGACCAGCCACACTGGGACTGAGACACGGCCCAGACTCCTACGGGAGGCAGCAGTGGGGAATTTTTCGCA<br>ATGGGGGCAACCCTGACGAAGCAATGCCGCGTGGATGAAGAAGGCCTTCGGGTTGTAAAGTCCTTTCGTGGGGGACG<br>AAAAGGCGGGTCCTAATACGATCTGCTGTTGACGTGAACCCAAGAAGAAGCACCGGCTAACTCCGTGCCAGCAGCCG<br>CGGTAATACGGGGGGTGCAAGCGTTAATCGGAATCACTGGGCGTAAAGGGTGCGTAGGCGGTACGTTAGGTCTGTCG<br>TGAAATCCCCGGGCTCAACCTGGGAATGGCGGTAGAAACCGGCGCACTAGAGTATGGGAGAGGGTGGTGGAATTCCT<br>GGTGGAGCGGTGAAATGCGTAGAGATCAGGAGGAACACCAGTGGCGAAGGCGGCCACCTGGCCCAATACTGACGCTG<br>AGGCACGAAAGCGTGGGGAGCAAACAGGATTAGATACCCTGGTAGTCCACGCCCTAAACGATGAATACTAGATGTTT<br>GGTACCTAGCGTACTGAGTGTCGTAGCTAACGCGATAAGTATTCCGCCTGGGAAGTACGGCCGCAAGGTTAAAACTCA<br>AAGGAATTGACGGGGGCCCGCACAAGCGGTGGAGCATGTGGTTTAATTCGATGCAACGCGAAGAACCTTACCTGGGC<br>TTGACATGTCCGGAATTCTGCAGAGATGCGGGAGTGCCCTTCGGGGAATCGGAACACAGGTGCTGCATGGCTGTCGTC<br>AGCTCGTGTCGTGAGATGTTGGGTTAAGTCCCGCAACGAGCGCAACCCTTGTCCTTAGTTGCCAGCGGTTCGGCCGGG<br>CACTCTAGGGAGACTGCCGGTGACAAACCGGAGGAAGGTGGGGATGACGTCAAGTCCTCATGGCCTTTATGTCCAGG<br>GCTACACACGTGCTACAATGGCGCGTACAGAGGGAAGCCAAGCCGCGAGGTGGAGCAGACCCCAGAAAGCGCGTCGT<br>AGTTCGGATTGCAGTCTGCAACTCGACTGCATGAAGTCGGAATCGCTAGTAATCGCGGATCAGCATGCCGCGGTGAAT<br>ACGTTCCCGGGCCTTGTACACACCGCCCGTCACACCATGGGAGTGGATTGTACCAGAAGCAGCTAGCCTAACCTTCGG<br>GAGGGC	>**OTU_102**<br>TACGGGGGGTGCAAGCGTTAATCGGAATCACTGG<br>GCGTAAAGGGTGCGTAGGCGGTACGTTAGGTCTGT<br>CGTGAAATCCCCGGGCTCAACCTGGGAATGGCGG<br>TAGAAACCGGCGCACTAGAGTATGGGAGAGGTGC<br>GTGGAATTCCTGGTGGAGCGGTGAAATGCGTAGA<br>GATCAGGAAGAACACCCGTGGCGAAGGCGGCCAC<br>CTGGCCCAATACTGACGCTGAGGCACGAAAGCGT<br>GGGGAGCAAACAGG

S.thermosulfidooxidans DX2012	TGCAGTCGAGCGGACCTTCGGGTCAGCGGCGGACGGGTGAGGAACACGTGAGTGATCGGGCTGTGAGTGGGGGATAT<br>CGGGCCGAAAGGCGCGGCAATCCCGCATACGTTCCGGGGAACCGGAAGAAAGCTTGGCAACAGGCGCTCACAGGGG<br>AGCTCGCGGCCCATTAGCTAGTTGGGGGGGTAAGGGCCTCCCAAGGCGACGATGGGTAGCCGGCCTGAGAGGGTGAA<br>CGGCCACACTGGGACTGAGACACGGCCCAGACTCCTACGGGAGGCAGCAGTAGGGAATCTTCCACAATGGGCGCAAG<br>CCTGATGGAGCAACGCCGCGTGAGTGAAGACGGCCTTCGGGTTGTAAAGCTCTGTCTGTCGGGACGAAGACCGGCCC<br>GGAAGGGCCGGGGAGCCGGTACCGACGGAGGAAGCCCCTGCAAACTACGTGCCAGCAGCCGCGGTAAGACGTAGGG<br>GGCAAGCGTTGTCCGGAATTACTGGGCGTAAAGGGCGTGTAGGCGGTGCGATACGTAGCGGTTTTAAGCCTCCGGCTC<br>ACCCGGAGGAGGGCGGCTAAACGGTCGCGCTAGAGTACAGGAGAGGTGGGTGGAATTCCTGGTGGAGCGGTGAAATG<br>CGTAGAGATCAGGAAGAACACCCGTGGCGAAGGCGGCGCACTGGCCTGGCCCTGACGCTGAGGCGCGACAGCGTGGG<br>GAGCGAACGGGATTAGATACCCCGGTAGTCCACGCCGTAAACGATGGGTACTAGGTGTCGCCCGGGTCCACCGGGCG<br>GTGCCGGAGCTAACGCACTAAGTACCCCGCCTGGGGAGTACGGCCGCAAGGTTGAAACTCAAAGGAATTGACGGGGG<br>CCCGCACAAGCAGTGGAGCATGTGGTTTAATTCGACGCAACGCGCAGAACCTTACCAGGACTGGACACGCTCGTGAG<br>CGCCGCGAAAGCGGCGGGCCCTTCGGGGAGCGAGCGCAGGTGCTGCATGGTTGTCGTCAGCTCGTGTCGTGAGATGTT<br>GGGTTAAGTCCCGCAACGAGCGCAACCCTTGTCGTGTGTTGCCAGCGGTGCGGCCGGGCACTCACACGAGACTGCCGG<br>TGACAAACCGGAGGAAGGTGGGGATGACGTCAAATCCGCATGGCCTTGATGTTCTGGGCTACACACGTGCTACAATG<br>GTCCCGACAACGGGATGCGACGGCGCGAGCCGGAGCCAATCCTTCAAACGGGATCTCAGTTCGGATTGCAGGCTGCA<br>ACTCGCCTGCATGAAGCCGGAATTGCTAGTAATCGCCCATCAGCATGGGGCGGTGAATTCGTTCCCGGGCCTTGTACA<br>CACCGCCCGTCACACCACGAGAGTCGGCCACACCCGAAGCCGGGCGATCCAACCGCAGGTGCGG	**>OTU_121**<br>GACGTAGGGGGCAAGCGTTGTCCGGAATTACTGG<br>GCGTAAAGGGCGTGTAGGCGGTGCGATACGTAGC<br>GGTTTTAAGCCTCCGGCTCACCCGGAGGAGGGCG<br>GCTAAACGGTCGCACTAGAGTATGGGAGAGGGTG<br>GTGGAATTCCAGGTGTAGCGGTGAAATGCGTAGA<br>GATCAGGAAGAACACCCGTGGCGAAGGCGGCGCA<br>CTGGCCTGGCCCTGACGCTGAGGCGCGACAGCGT<br>GGGGAGCGAACGGG
